# A novel suicide shuttle plasmid for *Streptococcus suis* serotype 2 and *Streptococcus equi* ssp. *zooepidemicus* gene mutation

**DOI:** 10.1038/srep27133

**Published:** 2016-06-03

**Authors:** Rui Liu, Ping Zhang, Yiqi Su, Huixing Lin, Hui Zhang, Lei Yu, Zhe Ma, Hongjie Fan

**Affiliations:** 1 College of Veterinary Medicine, Nanjing Agricultural University, Nanjing, 210095, China; 2 Jiangsu Co-innovation Center for Prevention and Control of Important Animal Infectious Diseases and Zoonoses, Yangzhou 225009, China; 3 China Animal Health and Epidemiology Center, Qingdao, 266000, China

## Abstract

The *mariner*-based *Himar1* system has been utilized for creating mutant libraries of many Gram-positive bacteria. *Streptococcus suis* serotype 2 (SS2) and *Streptococcus equi* ssp. *zooepidemicus* (SEZ) are primary pathogens of swine that threaten the swine industry in China. To provide a forward-genetics technology for finding virulent phenotype-related genes in these two pathogens, we constructed a novel temperature-sensitive suicide shuttle plasmid, pMar4s, which contains the *Himar1* system transposon, TnYLB-1, and the *Himar1* C9 transposase from pMarA and the *repTAs* temperature-sensitive fragment from pSET4s. The kanamycin (Kan) resistance gene was in the TnYLB-1 transposon. Temperature sensitivity and Kan resistance allowed the selection of mutant strains and construction of the mutant library. The SS2 and SEZ mutant libraries were successfully constructed using the pMar4s plasmid. Inverse-Polymerase Chain Reaction (Inverse-PCR) results revealed large variability in transposon insertion sites and that the library could be used for phenotype alteration screening. The thiamine biosynthesis gene *apbE* was screened for its influence on SS2 anti-phagocytosis; likewise, the *sagF* gene was identified to be a hemolytic activity-related gene in SEZ. pMar4s was suitable for mutant library construction, providing more information regarding SS2 and SEZ virulence factors and illustrating the pathogenesis of swine streptococcosis.

Transposable elements (TEs), or transposons, are remarkably diverse molecular tools for random mutagenesis in bacterial chromosomes. Transposon-based, signature-tagged mutagenesis in bacteria is a widely used and effective strategy for finding new virulence factors and studying bacterial pathogenesis. This technique has pinpointed many genes that are crucial for the infectivity of a variety of pathogens^1^,^2^. Several transposon-based gene delivery systems have already been utilized to create mutant libraries in streptococci^3^,^4^, although almost all of them have properties that limit their usefulness. Tn*916* is a conjugative transposon in Gram-positive bacteria, but harbors a preferred insertion site of a conserved AT-rich sequence^5^. Tn*917* is available for high integration efficiency mutagenesis in many Gram-positive bacteria, but random mutants are relatively scarce due to the existence of hot spots, and as such, Tn*917* is less productive^2^. Many new transposons have been developed through modification of Tn*916*, such as Tn*3872* and Tn*3704*. These new transposons have been used for streptococcus mutation in many studies, but insertion site hot spots limit the application of these transposons^6^,^7^. Tn*10*, Tn*4001* and Mu-based transposons are rarely used in streptococcus.

*Himar1* is a transposable element that belongs to the *mariner* family of transposons. Originally isolated from *Haematobia irritans, Himar1* has been extensively used to generate large numbers of bacterial insertion mutants^8^,^9^,^10^,^11^,^12^,^13^,^14^,^15^,^16^. Due to its ubiquitous dinucleotide target, TA, and simple transposition mechanism (no obvious host factors required), *Himar1* has become the state-of-the-art genetic tool for random mutagenesis in bacterial genomes^17^. The *Himar1* system has successfully been used for the mutagenesis of *Streptococcus pneumoniae* and *Streptococcus equi* subsp. *equi*^18^,^19^. However, this system has never been used in *Streptococcus suis* serotype 2 (SS2) and *Streptococcus equi* ssp. *Zooepidemicus* (SEZ). Thus, the development of a novel *Himar1* transposon mutagenesis system suitable for these swine streptococcosis pathogens was the primary purpose of this study.

SS2 and SEZ are responsible for great economic losses to pig agriculture in China. These two pathogens are also capable of infecting human beings, thereby threatening public health^20^,^21^,^22^. Knowledge of the virulence factors of SS2 and SEZ is limited, restricting the study of their pathogenesis. Although previous work has determined the complete genome sequence of several SS2 and SEZ strains, most of their genes have unknown functions and remain uncharacterized^23^,^24^. Transposons are routinely employed to screen for genes related to a specific phenotype to investigate bacterial virulence genes. In this study, we constructed a temperature-sensitive plasmid with the *Himar1* system that can be used to generate mutants in the SS2 and SEZ genomes. Furthermore, we successfully constructed SS2 and SEZ mutation libraries, which are suitable for further virulence gene screening.

## Results

### Analysis of transcription factor *σ* ^A^ in SS2 and SEZ

**Figure 1:**
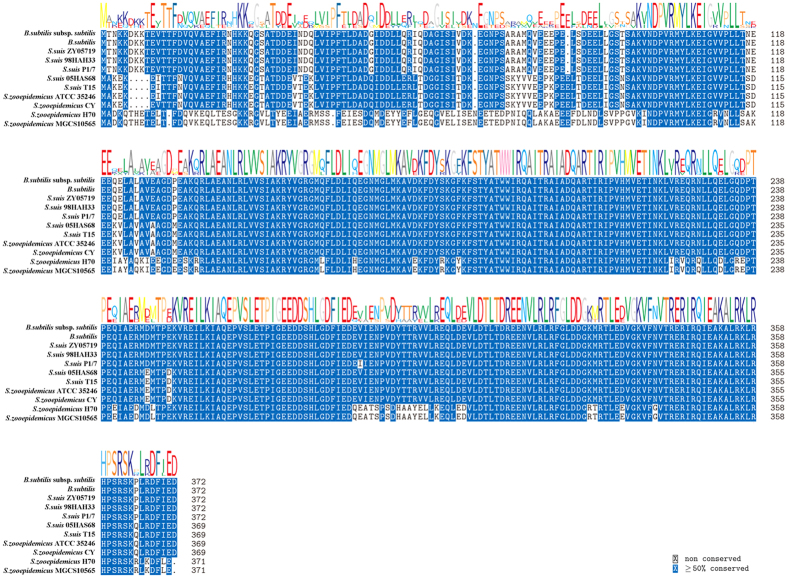
Homology analysis of the SigA protein from *B. subtilis* and the RopD protein from SS2 and SEZ. Position weight matrix (PWM) of each amino acid is shown with the alignment results. Amino acids with greater than 50% conservation are shown in blue. RopD from five SS2 strains and four SEZ strains were chosen to compare with the SigA of *Bacillus subtilis*.

The RopD in SS2 and SEZ has high homology to the SigA of *Bacillus subtilis,* which also known as *σ*^A^ with an active *Himar1* promoter. RopD from five SS2 strains and four SEZ strains were chosen to compare with the SigA in *Bacillus subtilis*. As shown in Fig. 1, there was greater than 90% homology among *B. subtilis* SigA protein, SS2 RopD protein and SEZ RopD protein. These proteins had high level of identity. Thus, we decided to retain the *Himar1* promoter of pMarA in the constructed pMar4s plasmid.

### Construction of the *mariner*-based pMar4s temperature-sensitive plasmid

Plasmid pMar4s was constructed for mutagenesis of SS2 and SEZ. pMar4s is a 8265 bp temperature-sensitive suicide plasmid containing a *repA*Ts fragment from the pSET4s plasmid. The TnYLB-1 transposon, which contains the ITR1 and ITR2 repetitive sequences and *kan* gene for kanamycin resistance, the *Himar1* C9 gene and its promoter were obtained from pMarA (Supplement 1). The pSET4s fragment was amplified by PCR with primers containing an *EcoR* I restriction enzyme cutting site; the pMarA fragment was obtained by direct digestion with *EcoR* I.

### Construction of SS2 and SEZ mutant libraries with pMar4s

pMar4s was used to alter the phenotypes of SS2 and SEZ to construct mutant libraries for use in selecting genes related to bacterial virulence (Supplement 2). Insertion of the TnYLB-1 transposon into the SS2 and SEZ genome was verified by PCR. As pMar4s contained Spc resistance on its backbone, loss of the plasmid from mutants was confirmed by culturing bacteria on Spc-resistance plates. Only PCR-positive, Kan-resistant, Spc-sensitive bacteria were included in the library. Of 275 randomly chosen SS2 mutants on the THB plates containing Kan, 193(70%) were Kan resistance and 82(30%) were Spc sensitivity. The transposition rate is about 70% in the SS2 mutants. In this manner, 2400 strains of SS2 mutants and 2400 strains of SEZ mutants were rapidly generated.

**Figure 2:**
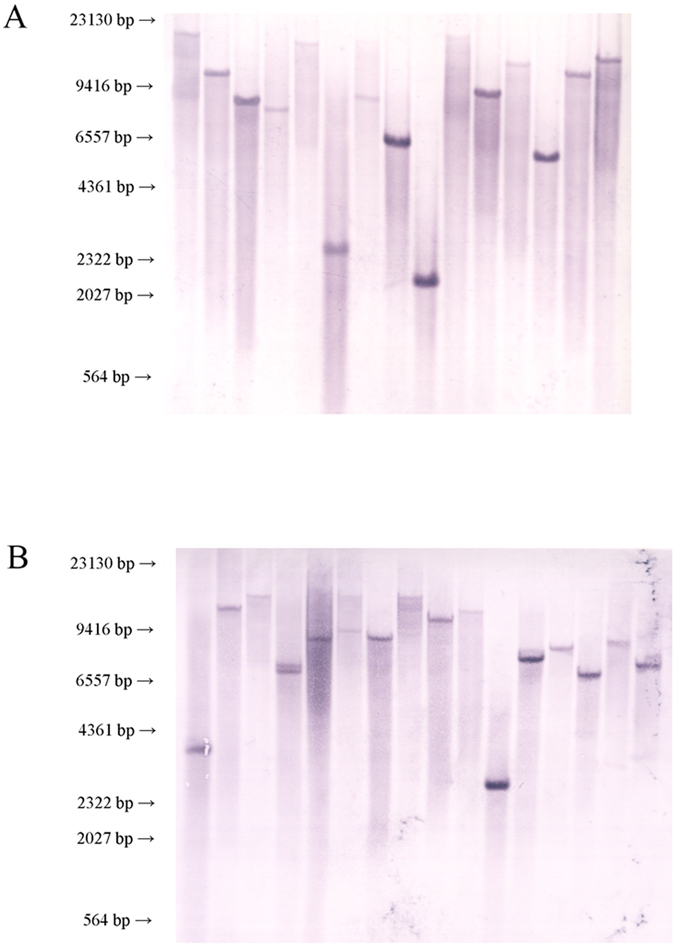
Southern blot analysis of the TnYLB-1 insertions in SS2 and SEZ. A 388 bp fragment of *kan* gene in TnYLB-1 was used as hybridization probe. (**A)** Genome of 15 SS2 transposon mutants were isolated and detected by Southern Blot. (**B)** Genome of 16 SEZ transposon mutants were isolated and detected by Southern Blot. DNA fragment sizes (kbp) are indicated to the left and are based on λ-Hind III digest DNA Marker included in the separating gel.

Mutants were randomly chosen from the SS2 and SEZ libraries for insertion site randomness detection by Inverse-PCR. The technological process and Inverse-PCR results are shown in Supplement 3. This technique revealed that the TnYLB-1 transposon inserted in different locations of the bacterial genome, indicating high insertion site variability. PCR fragments of SS2 were then sequenced and analysised by the BLAST program (Table 1). Southern Blot results verified the conclusion of Inverse-PCR. A 370 bp fragment of *kan* gene in TnYLB-1 was used as hybridization probe, the primers used in amplification of this fragment was listed in Table 2.SS2 and SEZ mutant libraries both had great insertion sites diversity (Fig. 2).

### Characterization of SS2 transposon mutant genes exhibiting an altered phenotype on anti-phagocytosis

**Figure 3:**
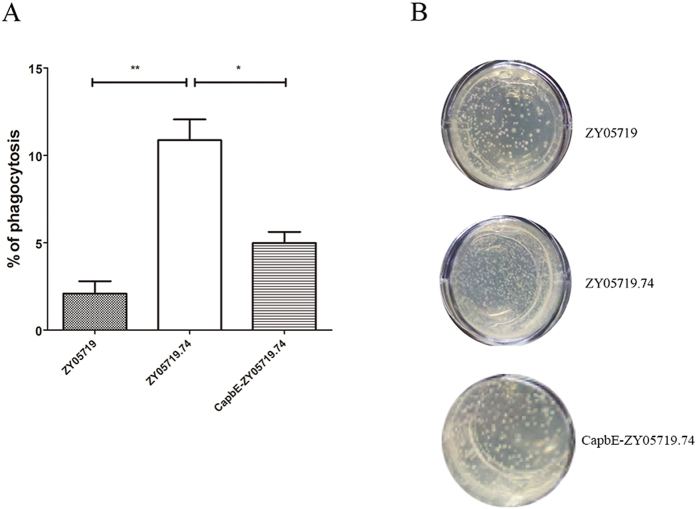
Identification of an SS2 transposon mutant exhibiting an altered anti-phagocytosis phenotype. (**A**) ZY05719.74 mutant showed a significant decrease in its anti-phagocytosis phenotype compared to the ZY05719 wild strain (*t*-test statistical analysis). The apbE gene complement strain CapbE-ZY05719.74 restored the anti-phagocytosis phenotype. (**B**) The screening results of ZY05719.74 in 24-well plates. BV2 cells were cultured in 24-well plates; SS2 was used to infect cells with MOI = 1:1; after 1 h of incubation, extracellular bacteria was kill by 10 μg/ml penicillin G and 100 μg/ml gentamycin; ddH_2_O was added to make cells lysis; 1 ml THB containing 7.5% agar was added to each well; bacterial number should be counted after culturing at 37 °C overnight.

The SS2 transposon mutant library was used to screen for genes related to bacterial anti-phagocytosis. After screening approximately 200 SS2 mutants, 1 mutant strain with decreased anti-phagocytosis ability was isolated and marked as ZY05719.74. This mutant was easily ingested by macrophages (Fig. 3). The transposon insertion site of this mutant was identified by determining the sequence flanking the TnYLB-1 transposon. Sequence data from the mutant was used to search the complete open reading frames of SS2. BLAST results showed that the thiamine biosynthesis protein *apbE* gene (GI:502375556) was inserted by the TnYLB-1 transposon. The *apbE* gene complement strain CapbE-ZY05719.74 restored the anti-phagocytosis phenotype. Though the phagocytosis rate was not as low as the wild type SS2, but it significantly decreased because of the *apbE* gene complementing in ZY05719.74.

### Characterization of SEZ transposon mutant genes exhibiting altered hemolytic activity

**Figure 4:**
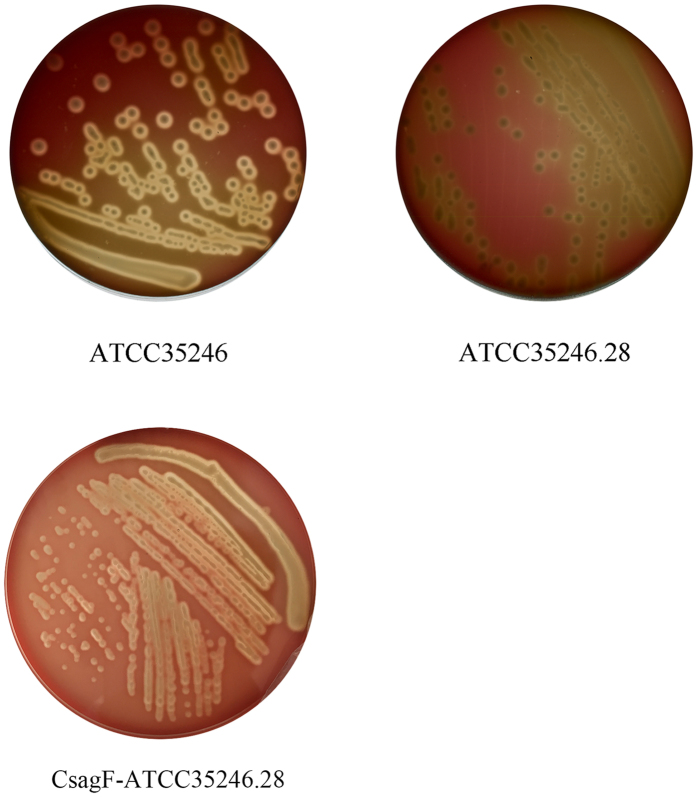
Identification of an SEZ transposon mutant exhibiting an altered hemolytic activity phenotype. The ATCC35246.28 SEZ mutant showed an altered hemolytic phenotype; the hemolytic activity of mutant strain decreased and exhibited alpha-hemolysis instead of beta-hemolysis on the sheep blood plates. The *sagF* gene complement strain CsagF-ATCC35246.28 restored the β-hemolytic phenotype.

The SEZ transposon mutant library was used to screen for genes related to bacterial hemolytic activity. Of the 300 SEZ mutants screened, 1 strain with an altered hemolytic phenotype was isolated and marked as ATCC35246.28. This strain showed alpha-hemolysis instead of beta-hemolysis to hemolyze sheep blood (Fig. 4). After amplifying and sequencing the sequence flanking the TnYLB-1 transposon, the membrane protein gene (GI:504435324), homology to streptolysin associated protein *SagF* gene in *Streptococcus pyogenes* MGAS10270 (GI:195974341), was identified as that inserted by TnYLB-1. This gene was separated by a gene homology to *sagE* gene of *Streptococcus pyogenes* MGAS10270 (GI:195974340) from *sagD* gene (GI:504435322) of SEZ. So we believed that this membrane protein gene was the *sagF* gene of SEZ. The *sagF* gene complement strain CsagF-ATCC35246.28 was constructed and it restored the hemolytic phenotype as showing in Fig. 4.

## Methods and Materials

### Cells, bacterial strains and growth conditions

The SS2 strain, ZY05719, and the SEZ strain, ATCC 35246, were isolated from dead pigs in the Sichuan province of China. The bacteria were cultured in Todd Hewitt broth (THB) (Oxoid Ltd., USA). Solid media contained 1.5% agar. When necessary, antibiotics were added to the plate or broth at the following concentrations: ampicillin (Amp), 100 μg/ml; spectinomycin (Spc), 50 μg/ml; kanamycin (Kan), 50 μg/ml. Hemolysis-deficient SEZ mutants were identified as colonies unable to show beta-hemolysis on THB plates containing 5% defibrinated sheep blood. The *Escherichia coli* strain DH5α was used in this study. It was cultured in Luria-Bertani (LB) medium. When pMar4s was transferred into *E. coli*, the cultural condition was 37 °C, antibiotics were added to the plate or broth at the following concentrations: spectinomycin (Spc), 50 μg/ml; kanamycin (Kan), 50 μg/ml. The microglial BV2 cell line was purchased from the American Type Culture Collection (ATCC) and cultured in DMEM (Gibco, USA) containing 10% fetal bovine serum (FBS) (Gibco, USA).

### Construction of the pMar4s plasmid

The temperature-sensitive shuttle plasmid pMar4s was derived from pMarA^25^ and pSET4s^26^. The fragment containing the temperature-sensitive replication origin of pWV01 lineage and the Spc-resistance gene of pSET4s plasmid was amplified using the primers ST1 and ST2 (Table 2), and *Eco*RI restriction sites were added to both ends of the PCR product. pMarA was digested with *Eco*RI and the 5.4 kbp fragment containing the *mariner*-based TnYLB-1 transposon was extracted from the gel and ligated to the fragment from pSET4s to construct the pMar4s plasmid.

### Competent bacterial cell preparation and electroporation

The pMar4s plasmid was transformed into SS2 and SEZ by electroporation^27^,^28^. Briefly, the optical density of the bacterial culture medium at 600 nm (OD_600_) was monitored with a spectrophotometer. SS2 was cultured in THB with the addition of 40 mM DL-threonine to an OD_600_ = 0.4. SEZ cells were prepared similarly but were treated with hyaluronidase Type IV (Sigma, USA) (45 U/ml for 30 min) to allow efficient pelleting. The cells were harvested by centrifugation at 10,000 × *g*, the supernatant was discarded, and the pellets were washed twice in 20 mL of 0.5 M sucrose solution. Finally, the cells were resuspended in 250 μL of a 10% glycerol and 0.5 M sucrose solution, and divided into aliquots. The competent cells were stored at −80 °C.

Approximately 1 μg of the pMar4s plasmid was added to the competent cells. The electroporation parameters were as follows: 25 kV/cm, 200 Ω and 25 μF. Immediately after electric shock, pre-warmed THB media containing 0.3 mmol glucose (1 mL) was added to the cuvette and incubated at 28 °C for 3–4 h.

### Detection of transposition events and mutant library construction

After recovery at 28 °C, the transformed colonies were spread on THB agar plates containing Kan and cultured at 37 °C for 24–48 h. The clones that had Kan resistance and Spc sensitivity were chosen for further analysis. To verify that these clones contained the TnYLB-1 transposon, they were subjected to PCR analysis using the oITR primer^25^. Positive colonies were selected as transposants and used to construct the mutant library.

### Identification of altered SS2 and SEZ phenotypes

For SS2, the anti-phagocytosis phenotype was chosen for screening in the SS2 mutant library. BV2 cells were cultured in 24-well plates. The MOI of SS2 to BV2 was 1:1. After incubating SS2 with BV2 for 1 h, SS2 was washed off with PBS 3 times. 100 mg/ml penicillin G was used to sterilize the extracellular bacteria. After washing with PBS 3 times to remove the penicillin G, 100 μl ddH_2_O was added to the 24-well plates to disrupt the BV2 cells and release the ingested SS2. Liquid THB containing 0.75% agar was added to the 24-well plates and cultured at 37 °C overnight. Clones with high growth density in the 24-well plates were selected for further analysis.

The SEZ mutant library was screened for hemolytic activity phenotype. Mutants of the library were spread on THB plates containing 5% defibrinated sheep blood. Clones lacking hemolytic activity were selected for further analysis.

### Mapping of transposon insertion sites

Transposon insertion sites were mapped as described by Le Breton with some modifications^25^. Mutant bacterial genomic DNA was digested with *TaqI* and ligated end to form a circle with a Rapid Ligation Kit (Roche, USA). The ligation products were purified and used as the template for inverse-PCR. The C1 and C2 primers^25^ used in Inverse-PCR are listed in Table 2. Inverse-PCR products were purified using the QIAgen PCR purification kit (Qiagen, USA) and sequenced with the oIPCR primer^25^ (Table 2). PCR fragments were sequenced by the Shanghai Sunny Biotechnology Company Limited. DNA sequence analyses were performed using the BLAST progrom (http://www.ncbi.nlm.nih.gov/BLAST/).

Southern blot analyses were performed using a DIG High Prime DNA labeling and detection starter kit I (Roche, USA). Briefly, a 388 bp fragment of *kan* gene in TnYLB-1 was used as hybridization probe, the primers used in amplification of this fragment was listed in Table 2. Genome of 15 SS2 transposon mutants and genome of 16 SEZ transposon mutants were randomly isolated and digested with E*coR* I, then used for Southern Blot analysis.

### Bioinformatics analysis

Multiple sequence alignment of transcription factor *σ*^A^ among *Bacillus subtilis*, SS2 and SEZ was performed using the msa package of the R statistical computing platform^29^. Plasmid sequence analyses were performed using Vector NTI Advance 11.0 (Invitrogen, USA). The BLAST program (http://www.ncbi.nlm.nih.gov/BLAST/) was used for database analysis.

### Construction of complemented strains

In this stduy, the promoter sequence of the IMPDH^30^ was used for the construction of the two complement plasmids. As we constructed the two complementation plasmids with the promoter sequence and the target genes sequences by using splicing-by-overlap-extension(SOE) PCR^31^, the two promoter sequences were amplified from SS2-ZY05719 genomic DNA by PCR using the primers RP1/R2 and RP1/P2. The sequences of target genes were amplified from SS2-ZY05719 and SEZ-ATCC 35246 genomic DNA respectively by PCR using the primers in the Table 2. Then the promoter sequence and the target genes sequences were fused by SOE PCR. After digestion with *Sph* I and *EcoR* I, the framents were cloned into shuttle vector pSET2^32^. These two vectors were transformed into the ZY05719.74 and ATCC35246.28 respectively to obtain the complement strains CapbE-ZY05719.74 and CsagF-ATCC35246.28^26^.

### Ethics

We confirmed that all experiments were performed in accordance with relevant guidelines and regulations of the Science and Technology Agency of Jiangsu Province. The Nanjing Agricultural University Veterinary College academic board approved all experiments in this research.

## Discussion

In China, swine streptococcosis is responsible for enormous economic loss and threatens the pig farming industry^33^. Many humans have died from infections with pathogens of swine streptococcosis since 1977. SS2 and SEZ have been identified as the most important pathogens that can cause swine streptococcosis^34^. Though many virulence factors have been discovered in these two bacteria through genomic sequence and bioinformatics analyses, there has not been much progress in determining the pathogenesis of these two bacteria^35^. Because reverse genetics technologies have shown limitations to precisely determine the genes related to virulent phenotypes from large datasets. As such, the development of an effective forward genetics virulence gene location technology was the primary purpose of this study.

Though the *mariner* family of transposons had been used in Gram-positive bacterial mutants such as *Streptococcus mutans* and *Streptococcus pyogenes*^36^,^37^,^38^,^39^ for many years, but this system has not previously been used to study swine streptococcosis pathogens. *Himar1* has a ubiquitous dinucleotide target, TA, and requires no species-specific host factors^40^. As its insertion sites are more random than many other transposons, *Himar1* has become a great genetic tool for random mutagenesis in Gram-positive bacterial genomes and has recently been utilized to create mutant libraries of prokaryotes.

In this study, we used pMarA and pSET4s to construct a new plasmid, pMar4s, which could be used in mutating SS2 and SEZ. As *σ*^A^, the transcription factor that binds to the promoter of transposase *Himar1* C9, was highly homologous among *B. subtilis*, SS2 and SEZ, we retained this promoter in pMar4. The subsequent results prove that this promoter was universal in these 3 different bacteria. The *repA*Ts fragment imparted temperature sensitivity to pMar4s in SS2 and SEZ. This plasmid could only tolerate a 28 °C culturing condition; pMar4s was lost when the temperature was set to 37 °C. The Amp and Spc resistance genes lost their efficacy; only the Kan resistance gene inserted into the genome with the transposon retained its function. This design simplified the screening process.

Both the SS2 and SEZ mutant libraries were constructed successfully and could be used for phenotype screening. Inverse-PCR technology was able to detect the sequence flanking the TnYLB-1 transposon insertion site. Inverse-PCR also revealed the randomness of the transposon insertion site and Southern Blot results verified these results. The sampling results showed that the SS2 and SEZ mutant libraries both had great variety and were appropriate for phenotype screening.

We chose an anti-phagocytosis phenotype for the SS2 mutant library screening and a hemolytic activity phenotype for the SEZ mutant library screening. In the SS2 altered anti-phagocytosis phenotype mutant, TnYLB-1 inserted into the thiamine biosynthesis gene, *apbE*. Thiamine is an important nutrient for bacterial capsular polysaccharide (CPS) synthesis^41^. CPS helps pathogens evade ingestion by macrophages^42^. Inhibiting the biosynthesis of thiamine might influence CPS formation in SS2. Although the *apbE* gene might not be directly related to anti-phagocytosis in SS2, its mutant indeed altered the anti-phagocytosis phenotype by affecting a bacterial physiological process. Alternatively, there might be a novel mechanism connecting thiamine biosynthesis and SS2 anti-phagocytosis that needs to be studied further. The *sagF* gene was identified as the TnYLB-1-inserted gene in the SEZ hemolytic activity-altered phenotype mutant. In our previous study, the *sagD* gene was identified as a virulence factor and its mutation led to the loss of SEZ hemolytic activity^43^. The *sagF* gene was located in the same operon with *sagD* gene in SEZ genome, it should play an important role in SEZ hemolytic activity and was an indispensable gene in sag operon^44^.

In conclusion, we constructed pMar4s with the pMarA and pSET4s backbone plasmid. This novel plasmid, pMar4s, was effective for mutating SS2 and SEZ, and its insertion site variety was suitable for constructing SS2 and SEZ mutant libraries for use in altered phenotype screening. This forward genetics virulence gene location technology will provide more information regarding SS2 and SEZ virulence factors and help to illustrate the pathogenesis of swine streptococcosis.

## Additional Information

**How to cite this article**: Rui, L. *et al.* A novel suicide shuttle plasmid for *Streptococcus suis* serotype 2 and *Streptococcus equi* ssp. *zooepidemicus* gene mutation. *Sci. Rep.* **6**, 27133; doi: 10.1038/srep27133 (2016).

## Supplementary Material

Supplementary Information

## Figures and Tables

**Table 1:** DNA sequence analysis of SS2 insertional mutants obtainen by TnYLB-1 tansposition from pMar4s.

Stain	Transposon insertion	Gene name
ZY05719.35	ZY05719_RS02345	ribonuclease G
ZY05719.120	ZY05719_RS02540	permease
ZY05719.235	ZY05719_RS07110	phosphoglycolate phosphatase
ZY05719.456	ZY05719_RS03750	branched-chain amino acid ABC transporter substrate -binding protein
ZY05719.555	ZY05719_RS06850	type I restriction endonuclease subunit S
ZY05719.631	ZY05719_RS08285	1,4-dihydroxy-2-naphthoate octaprenyltransferase
ZY05719.818	ZY05719_RS06845	ribose 5-phosphate isomerase
ZY05719.999	ZY05719_RS06545	ABC transporter permease
ZY05719.1146	ZY05719_RS09300	DNA mismatch repair protein MutT
ZY05719.1313	ZY05719_RS00990	AraC family transcriptional regulator
ZY05719.1553	ZY05719_RS09965	recombinase RarA
ZY05719.1721	ZY05719_RS02070	16S rRNA methyltransferase
ZY05719.1818	ZY05719_RS02315	asparagine synthetase AsnA
ZY05719.1933	ZY05719_RS02005	penicillin-binding protein
ZY05719.2000	ZY05719_RS03105	hypothetical protein

**Table 2:** Primers used in this study.

Primer name	Sequence (5′-3′)	Restriction Enzyme Site
ST1	ATCATCGAATTCACTAGTGTTCGTGAATACATGTTATAATAACTA	*EcoR*I
ST2	ATCATCGAATTCAGATCTATTAATCGCAACATCAAAC	*EcoR*I
oIPCR	GCATTTAATACTAGCGACGCC	
C1	GCTTGTAAATTCTATCATAATTG	
C2	AGGGAATCATTTGAAGGTTGG	
Southern-F	TGATCCCCAGTAAGTCAAAAAA	
Southern-R	TGCATCAGGCTCTTTCACTCCA	
RP1	ACATGCATGCAAAGAAAGCATTTACATA	SphI
R2	ACGAAGGGTAATAATGTAGCAT AACTGTCTTCCTGTAATA	
*apbE* -F	TATTACAGGAAGACAGTT ATGCTACATTATTACCCTTCGT	
*apbE* -R	CCGGAATTCTTAAGGCATTGGGTGTACAAAGGC	*E*co*R*I
P2	TGCTAGTATTATGATCATGTTCTTTCCTTTCTTTTGGG	
sagF-F	CCCAAAAGAAAGGAAAGAACATGATCATAATACTAGCA	
sagF-R	CCGGAATTCCTAATGCTGCTCTTTAAAACTAAT	*E*co*R*I
