# Supplementary material for: A novel suicide shuttle plasmid for Streptococcus suis serotype 2 and Streptococcus equi ssp. zooepidemicus gene mutation
Source: Sci Rep. 2016 Jun 3;6:27133. doi: 10.1038/srep27133 (PMC4891806; doi:10.1038/srep27133)
Supplement: Supplementary Information [file srep27133-s1.pdf]

A novel suicide shuttle plasmid for *Streptococcus suis* serotype 2 and *Streptococcus equi* ssp. *zooepidemicus* gene mutation

Liu Rui[a], Zhang Ping[a], Su Yiqi[a], Lin Huixing[a,b], Zhang Hui[c], Yu Lei[a], Ma Zhe*[a,b] and Fan Hongjie[a,b]

Supplement 1

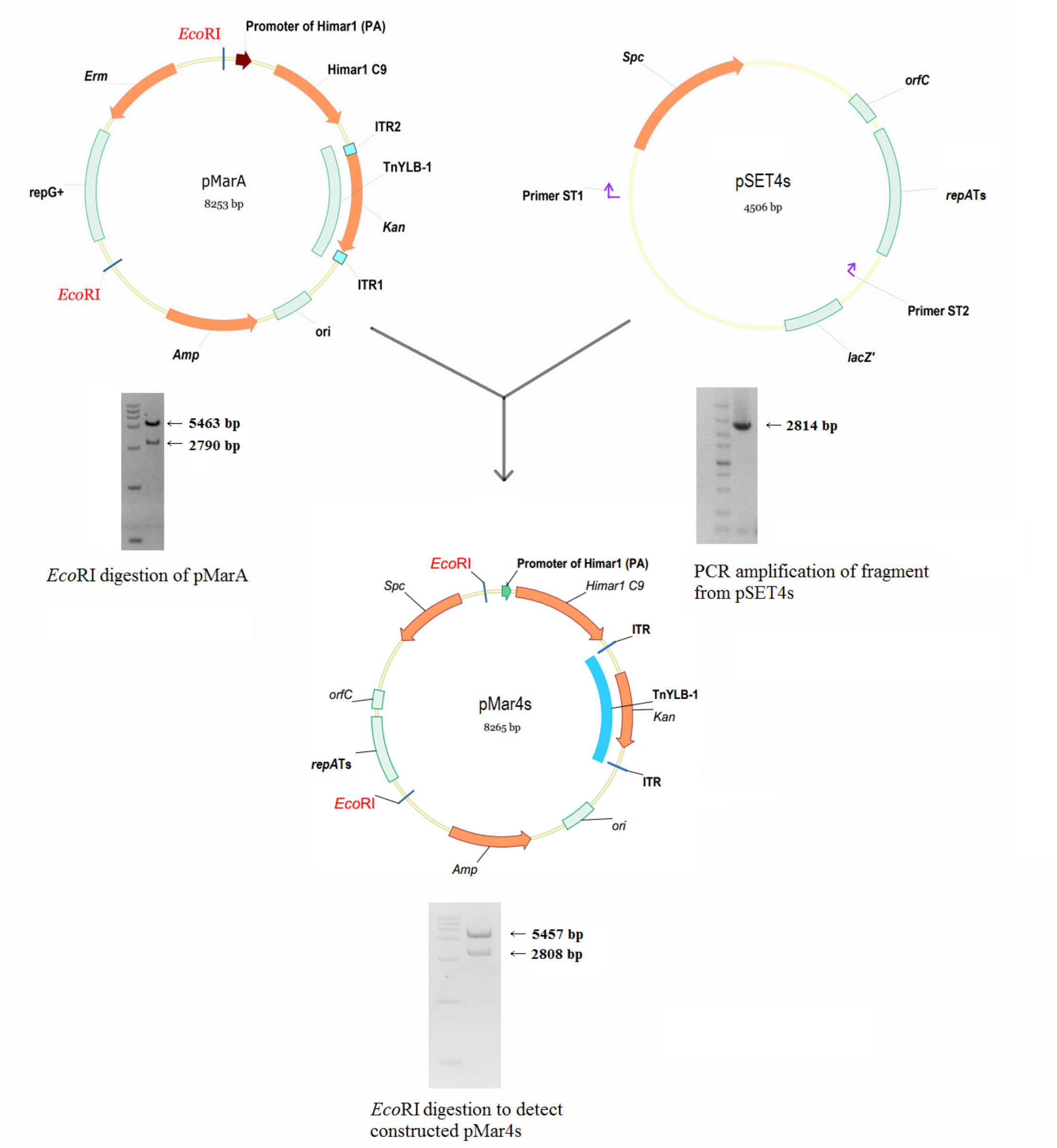

Supplement 1. Construction diagram of the temperature-sensitive suicide plasmid pMar4s. The *repA*Ts temperature-sensitive fragment was PCR amplified from the pSET4s plasmid using ST1 and ST2 primers. The TnYLB-1 transposon and promoter of *Himar1* was digested from the pMarA plasmid with *Eco*RI. The constructed pMar4s plasmid was verified by *Eco*RI digestion.

Supplement 2

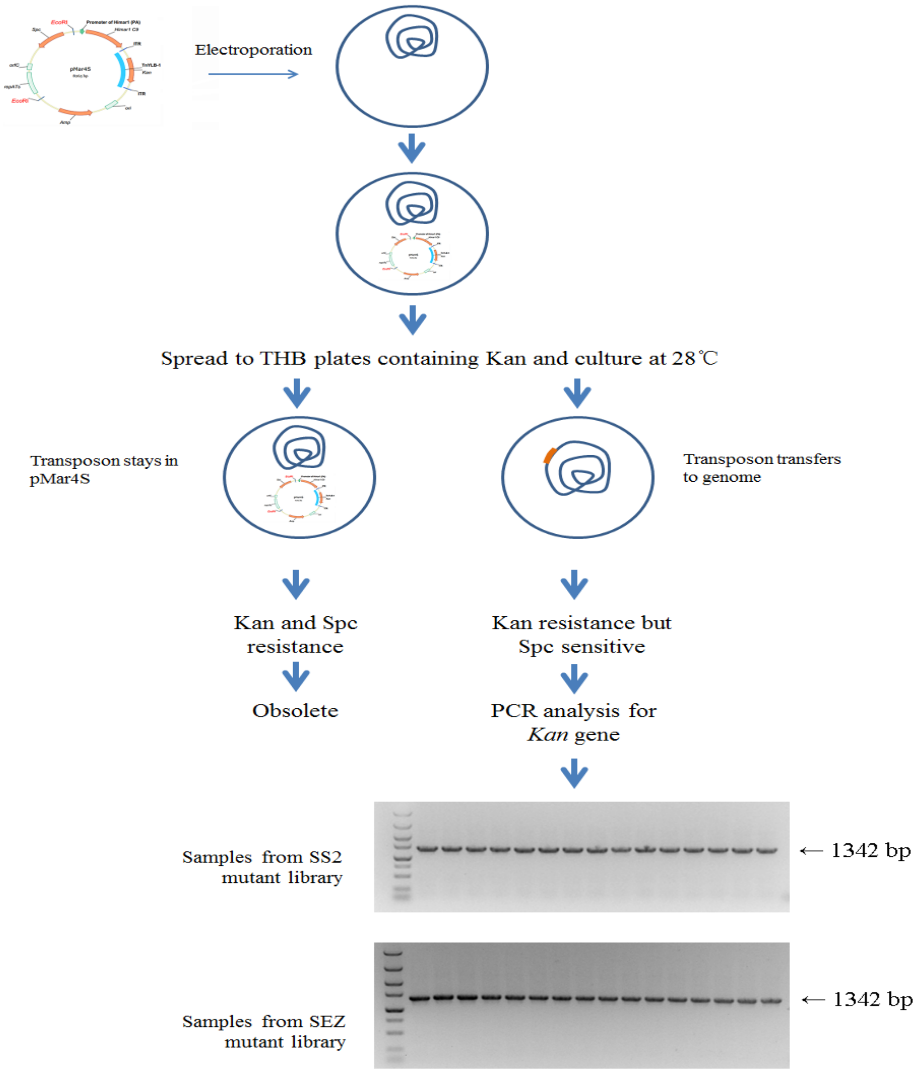

Supplement 2. Diagram of SS2 and SEZ mutant library construction with the pMar4s plasmid. The bacteria that had positive PCR results and were resistant to Kan but sensitive to Spc were included in the library.

Supplement 3

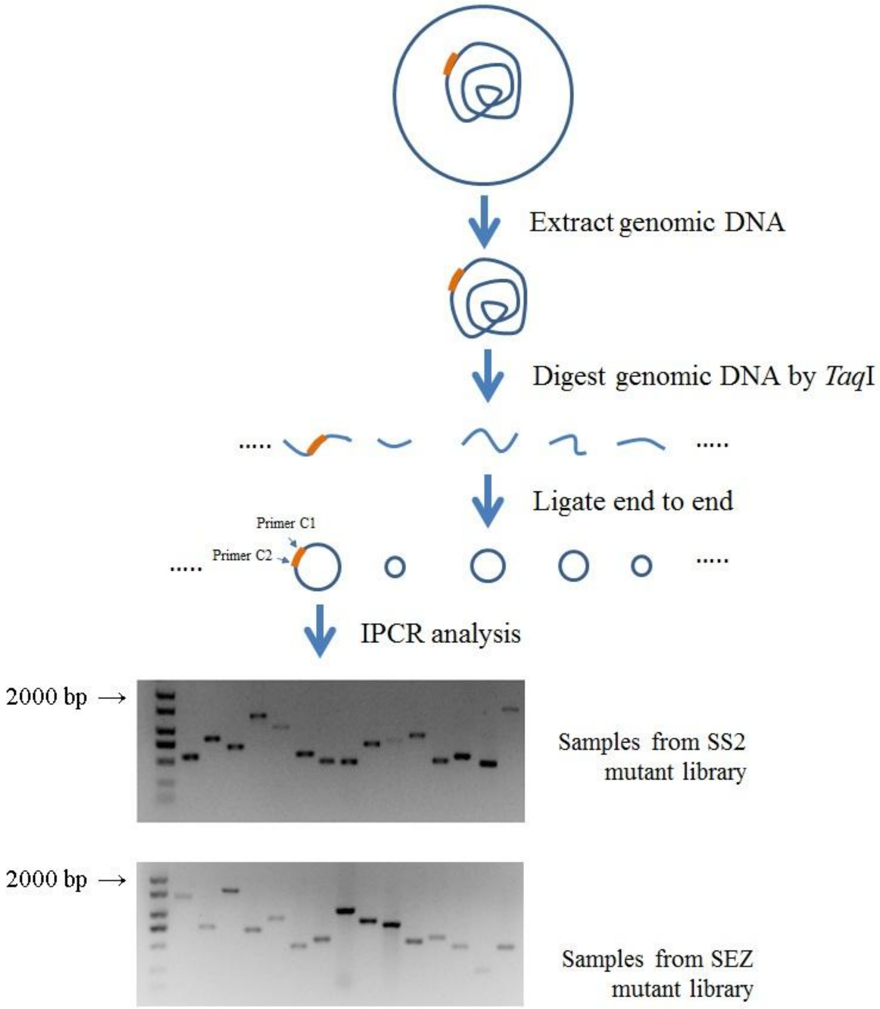

Supplement 3. The identification of the TnYLB-1 transposon insertion site. The bacterial genome was extracted and digested, and after self-ligation, the circle fragment was used for Inverse-PCR with the primers C1 and C2. The TnYLB-1 transposon was inserted in different locations of the bacterial genome, and the insertion sites had good variety.
